# Nonthermal fluctuations accelerate biomolecular motors

**DOI:** 10.1007/s12551-024-01238-x

**Published:** 2024-10-02

**Authors:** Takayuki Ariga

**Affiliations:** Graduate School of Frontier Biosciences, Osaka University, Suita, Japan

**Keywords:** Molecular motor, Kinesin, Single molecule manipulation, Optical tweezers, Nonthermal fluctuations

## Abstract

Intracellular transport is essential for maintaining cellular function. This process is driven by different mechanisms in prokaryotic and eukaryotic cells. In small prokaryotic cells, diffusion is the primary means of transport, while larger eukaryotic cells also rely on active transport by molecular motors such as kinesin and dynein. Recently, it has become evident that, in addition to diffusion based on thermal fluctuations (Brownian motion), which was conventionally considered a diffusion mechanism within living cells, nonthermal fluctuations generated by metabolic activities play a crucial role in intracellular diffusion. Similarly, while molecular motors have been proposed to exploit thermal fluctuations in the environment following the direct observation and manipulation of single molecules, they have also been reported to utilize nonthermal fluctuations in recent years. This review begins with a brief overview of the historical knowledge of diffusive intracellular transport, which has been extended from the thermal fluctuations to the nonthermal fluctuations generated by metabolic activity. It then introduces recent findings on how nonthermal fluctuations accelerate the motion of molecular motors and discusses future perspectives on the general effects of these fluctuations on molecules in living cells.

## Intracellular transport and fluctuations

**Fig. 1:**
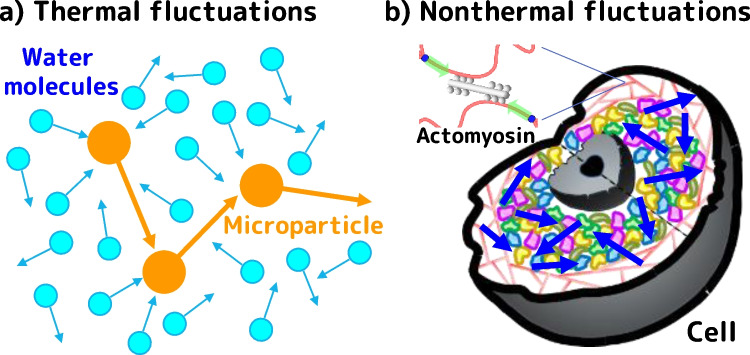
Thermal and nonthermal fluctuations. **a** Microparticles in water move in a random manner, pushed by the thermal motion of water molecules even at equilibrium conditions. **b** Inside living cells, the cytosol is actively agitated by the consumption of chemical free energy derived from metabolic activity

Intracellular transport mechanisms differ significantly between prokaryotic and eukaryotic cells. In prokaryotic cells, diffusion primarily drives transport, whereas in larger eukaryotic cells, vesicles are actively transported by biomolecular motors. These mechanisms are well-documented in standard textbooks (Phillips et al. 2011). However, it has recently been shown that not only thermal fluctuations, which were previously thought to be responsible for intracellular diffusion, but also nonthermal fluctuations are produced within living cells and actively influence intracellular transport (Fig. 1) (Guo et al. 2014; Parry et al. 2014). The most basic definition of nonthermal fluctuations is 'all fluctuations other than thermal fluctuations'; that is, fluctuations produced by active manipulation, such as externally applied force, stirring, or heating, and so on. Before discussing how they are generated in the cell and their physiological significance and role, let us first review the historical background of conventional thermal fluctuations.

Diffusion is a phenomenon that can be represented by how a single drop of ink in a glass of water gradually spreads and eventually forms a uniform, dilute solution. From a microscopic perspective, this process is based on the random motion of tiny particles and is known as "Brownian motion," which has been observed since the invention of the microscope at the end of the sixteenth century. Although some of the observed movements at that time were in fact caused by microorganisms (Leeuwenhoek 1677), it was initially believed that all such movement was a result of biological activity. Robert Brown, a 19th-century botanist, also held this belief while observing the random movement of tiny particles (Brown terms these particles as “molecules”) released when pollen burst in water. He expected the random movement to cease when the pollen's vital activity stopped and observed the movement under various lethal conditions. However, all his attempts to stop this movement failed, leading him to discover that even inorganic microparticles exhibit “*vivid*” movement (Brown 1828).

Brown's achievement lies in his discovery of the universality of random motion. Brownian motion occurs regardless of the type of material composing the microparticles and becomes more active as the particle size decreases, the temperature increases, or the viscosity of the solution decreases. Today, we know that Brownian motion is caused by collisions resulting from the thermal motion of numerous molecules (Einstein 1905; Perrin 1909), and thus it is also called "thermal fluctuation" (Fig. 1a). Since thermal fluctuations always occur even in equilibrium at any temperature above absolute zero, no external energy is needed for diffusion. In prokaryotic cells (around micrometer size), newly synthesized proteins can quickly diffuse to various locations within the cell. In contrast, membrane proteins synthesized near the nucleus in eukaryotic cells must be transported to the cell periphery while embedded in vesicles. In human neurons, for example, this distance can be up to 1 m. However, the time required for diffusion to cover such a distance increases proportionally to the square of the distance, making it impractical for cells to function and thus living organisms to survive. Instead, in eukaryotic cells, biomolecular motors such as kinesin, dynein and myosin actively transport vesicles using chemical free energy derived from ATP hydrolysis.

## Nonthermal fluctuations in cells

As mentioned earlier, it has long been believed that materials in prokaryotic cells diffuse solely through thermal fluctuations, requiring no external energy input. However, analysis of the diffusive motion of intracellular particles in *Escherichia coli* has revealed that this motion, which at first glance appears to be mere Brownian motion, becomes significantly suppressed when metabolic activity is inhibited (Weber et al. 2012; Parry et al. 2014). This phenomenon clearly has a different origin from the thermal fluctuation investigated by Brown. We thus refer to this phenomenon as "nonthermal fluctuations" (also known as “nonequilibrium fluctuations”) (Fig. 1b).

Prior to this observation of prokaryotic cells, nonthermal fluctuations dependent on metabolic activity had already been observed in eukaryotic cells (Caspi et al. 2000; Lau et al. 2003; Bursac et al. 2005; Wilhelm 2008; Gallet et al. 2009). Recent technical advancements have enabled quantitative measurements of these fluctuations (Fakhri et al. 2014; Guo et al. 2014; Nishizawa et al. 2017a; Hurst et al. 2021; Umeda et al. 2023). The magnitude of nonthermal fluctuations observed in eukaryotic cells is dramatically reduced by inhibiting the activity of intracellular myosin, suggesting that the fluctuations are mainly generated by the movement of myosin minifilaments dispersed throughout the cell (Fakhri et al. 2014; Guo et al. 2014). However, nonthermal fluctuations are not completely abolished by inhibiting myosin alone. Furthermore, they are also observed in prokaryotic cells that lack myosin and other molecular motors, indicating that other factors, such as the “enhanced diffusion” or “ballistic motion” of catalytically active enzymes (Muddana et al. 2010; Riedel et al. 2015; Jee et al. 2018), may be involved in the formation of nonthermal fluctuations. The details, however, remain controversial (Zhang and Hess 2019).

The origin of nonthermal fluctuations within cells is still under debate. However, they can be broadly classified into two categories. One is the hydrodynamic forces generated by the viscous coupling between the solvent and the directional movement of objects associated with motor proteins. As mentioned above, various origins can be considered, including not only cytoskeletal motors like actomyosin in eukaryotic cells but also transcription and translation systems in prokaryotic cells. Experimental and theoretical analyses of diffusion and hydrodynamic interactions in an intracellular crowded environment, using single-particle tracking, have been reviewed in this journal (Hall and Hoshino 2010). In recent years, fluid–structure interactions have been studied using various model systems, such as the active carpet model, which consists of microtubules and kinesins (Chakrabarti et al. 2024), and the active swimmer model, which considers microorganisms as actively moving particles (Zaid and Mizuno 2016; Kurihara et al. 2017; Kanazawa et al. 2020).

The other origin of nonthermal fluctuation is extra-thermal effects arising from local heating or cooling due to spatially heterogeneous exothermic or endothermic reactions. At present, however, it is challenging to discuss accurately because thermodynamic temperature, as a physical quantity, is defined only in homogeneous, equilibrium states. It should be noted that, in some cases, the concept of “effective temperature” is used as a measure to quantify nonthermal fluctuations (Cugliandolo et al. 1997; Fodor et al. 2016; Hayashi et al. 2018), but caution is needed as it tends to cause confusion with the effects of thermodynamic temperature change. This review will not address those details. Instead, it will primarily consider the impact of nonthermal fluctuations originating from the former origin on individual molecules in the following sections.

## Kinesin: a molecular motor for active transport

**Fig. 2:**
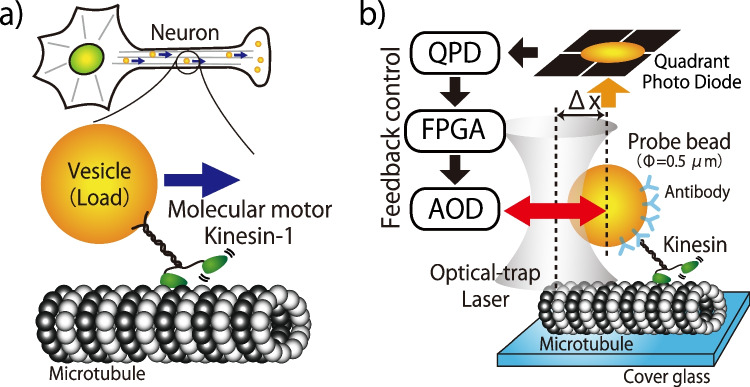
Kinesin and optical tweezers. **a** Molecular motor kinesin transports intracellular vesicles. **b** Schematic of the optical tweezers measurement system. Feedback control of the laser focus enables the application of arbitrary external forces to the walking kinesin via a probe particle

In eukaryotic cells, vesicle transport is carried out by molecular motors, such as myosin moving along actin filaments and kinesin and dynein moving along microtubules (Vale 2003; Hirokawa et al. 2009). Among them, kinesin-1 (or conventional kinesin; hereafter referred to as kinesin) is ubiquitously expressed in most cell types and is responsible for vesicle transport from the Golgi apparatus and endoplasmic reticulum to the cell periphery (Fig. 2a). The mechanism of kinesin movement has been investigated in detail by recently developed single-molecule measurement techniques. It has been revealed that kinesin transports cargo by alternately extending its two heads (ATP hydrolysis sites) along the microtubule tracks in a manner similar to bipedal walking (Yildiz et al. 2004; Mori et al. 2007). However, the two heads are connected by a flexible linker and cannot step out as precisely as human feet. Instead, the one head unbound from the microtubule during the walking fluctuates intensely due to Brownian motion. This thermal fluctuation of the floating head has been observed using single-molecule measurements with nanoscale gold particles (Mickolajczyk et al. 2015; Isojima et al. 2016). A model in which kinesin moves unidirectionally by selectively extracting forward motion by thermal fluctuations has long been proposed (Vale and Oosawa 1990) and actively discussed in recent years (Hwang and Karplus 2019).

Kinesin, which transports vesicles using the hydrolysis of ATP as an energy source, can also be viewed as an engine that converts chemical free energy into mechanical work. Its output work can be measured using optical tweezers (Fig. 2b). Optical tweezers is a technique in which a laser beam is focused to create a force that traps small objects towards its focus (Ashkin et al. 1986; Neuman and Block 2004) and was the subject of the 2018 Nobel Prize in Physics. Using this technique, when an external force is applied to a probe particle pulled by moving kinesin in the opposite direction to its motion, the kinesin is unable to move forward under a load of 6 to 7 pN (Svoboda and Block 1994; Kojima et al. 1997; Visscher et al. 1999), indicating the maximum force that kinesin can exert. Since kinesin advances 8 nm per ATP hydrolysis (Svoboda et al. 1993; Hua et al. 1997; Schnitzer and Block 1997), it produces about 50 pN·nm of maximum work per ATP. This value was estimated to give about 50% efficiency, given that the chemical free energy of ATP hydrolysis in the cell is about 100 pN·nm (Howard 2001). However, kinesin in the stalled state repeats back-and-forth steps while consuming free energy (Nishiyama et al. 2002; Carter and Cross 2005; Taniguchi et al. 2005). Therefore, the real efficiency of kinesin in the stall condition is zero.

Physiologically, kinesin functions as an intracellular vesicular transporter. Instead of performing work against an artificial external force, such as optical tweezers, kinesin in the cell continues to move while pulling loads within the intracellular environment (Fig. 2a). In this context, the chemical free energy utilized by kinesin should ultimately be dissipated to the environment as frictional heat through the load. Conventionally, this dissipation cannot be directly measured in such a fluctuating environment, but Harada and Sasa theoretically established a method to estimate dissipation through the measurement of fluctuations and responses (Harada and Sasa 2005). My colleagues and I previously quantified this dissipation under a microscope and found that it was dramatically small compared to the chemical free energy input, with as much as 80% of the energy being discarded instead of being transferred to the transport of the load (Ariga et al. 2018). This result is surprising, since kinesin appears to be an inefficient motor (Hendricks 2018). However, it is hard to believe that kinesins, which have evolved over hundreds of millions of years to transport cargo, suffer from such low efficiency. Therefore, we hypothesized that kinesins are not optimized for in vitro experimental conditions but ideally suited for the intracellular environment where they actually work (Ariga et al. 2020). Here, what I focused on as the difference between the two conditions is nonthermal fluctuation. Kinesin that walks using thermal fluctuations may also be successfully utilizing nonthermal fluctuations.

## Kinesin accelerates by external force fluctuations

**Fig. 3:**
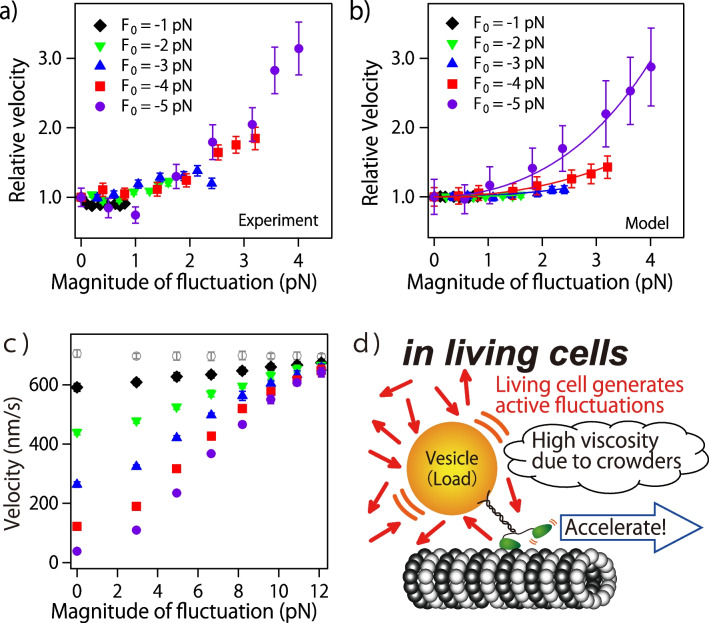
Acceleration of kinesin due to external force fluctuations. **a** Experimental results. **b** Numerical simulations using a mathematical model. **c** Numerical simulations with physiologically plausible external force fluctuations. **d** Acceleration phenomena with nonthermal fluctuations in living cells. Reprinted figures with permission from (Ariga et al. 2021). Copyright (2024) by the American Physical Society

To investigate the effects of intracellular nonthermal fluctuations on the motion of molecular motors, my colleagues and I developed an experimental system in which kinesin is subjected to external forces under a microscope that fluctuate as they do inside the cell (Fig. 2b) (Ariga et al. 2021). We numerically generated the nonthermal fluctuations observed inside cells, and by varying the distance between the laser focus of the optical tweezers and the probe bead attached to the tail of the kinesin, we applied the nonthermal force fluctuations to a probe bead in addition to a constant external force in the opposite direction of the kinesin’s motion. The measured velocity is shown in Fig. 3a as velocities normalized to the velocity without fluctuations at the same load. The horizontal axis of the figure shows the magnitude of the fluctuations as standard deviation, and each marker indicates the magnitude of the constant external force (load) excluding the fluctuation component. Especially in the region of high load, a tendency for kinesin to speed up with the magnitude of the fluctuation was observed. Although details of the mathematical model are omitted in this review (see our previous review (Ariga et al. 2020) for details), this acceleration phenomenon was reproduced by simulations using the model (Fig. 3b) and supports the conclusion that kinesin is accelerated by fluctuations in external forces.

As mentioned earlier, nonthermal fluctuations in eukaryotic cells are mainly produced by intracellular actin and myosin (Guo et al. 2014). Moreover, it has been reported that they can produce forces up to 30 pN (Kaya et al. 2017). In the kinesin experiment described above, the magnitude of the external force fluctuations was limited to the same level as the constant external force (a few pN) because the fluctuating external force was applied to kinesin by changing the focal position of the optical tweezers. However, with a mathematical model, there is no need to add such a restriction. Therefore, we performed a numerical simulation in which a fluctuating external force of up to 30 pN, which is physiologically plausible, was added to the mathematical model of kinesin (Fig. 3c). The results indicate that even when the kinesin speed is reduced by a large load, the nearly maximum speed of the unloaded condition can be achieved by a fluctuating external force.

The actual intracellular environment is not only characterized by nonthermal fluctuations but also by being a very crowded environment (Goodsell 1991; Hall and Hoshino 2010; Feig et al. 2017; Nishizawa et al. 2017b; Ebata et al. 2023). In such a crowded environment, viscosity is elevated, resulting in increased resistance to the vesicles being transported. A large constant external force applied under experimental conditions can be regarded as resistance in a crowded environment. This analogy suggests that kinesin actively utilizes nonthermal fluctuations generated within the cell, enabling smooth movement like that under the microscope, even in an intracellular crowded environment (Fig. 3d).

## Universality of acceleration phenomena due to fluctuations

So far in this review, I have avoided mathematical descriptions as much as possible, but this is not possible to understand the acceleration phenomenon described in the previous section. The Arrhenius equation shows that the logarithm of the reaction rate of an enzyme reaction is proportional to the inverse of the temperature. This empirical relationship is qualitatively explained as the larger the activation energy between the reactants and products, the longer it takes to overcome this energy barrier. The magnitude of the activation energy can be varied by applying an external force. The activation energy for the forward step of kinesin is increased by adding an external force *F* in the direction opposite of the kinesin movement (which is negative), and the reaction rate *k* for one forward step is slowed. Mathematically, this relationship is (Howard 2001)

1 $$k={k}^{0}\text{exp}(\frac{{d}_{f}F}{{k}_{B}T}),$$

where *k*^0^ is the rate constant at no load, *k*_B_ is Boltzmann's constant, *T* is the absolute temperature, and *d*_*f*_ is a parameter with distance dimension and represents the dependence on external force. Here, the rate constant *k* of the reaction process is described as an exponential function with the external force *F* as an argument.

On the other hand, the following Jensen’s inequality generally holds for functions with a concave shape, such as exponential functions (Jensen 1906).

2 $$\langle k(F)\rangle \ge k(\langle F\rangle ),$$

where < · > denotes the average value. In other words, the average reaction rate < *k* > when a fluctuating external force *F* is applied will always exceed the rate when the average value of the external force < *F* > is used as the argument. These two equations allow us to understand the phenomenon of kinesin acceleration due to a fluctuating external force.

An important consequence of mathematically describing the acceleration phenomenon is the predictive ability. The Arrhenius equation used here is an equation that holds for enzymatic reactions in general, in which case Jensen's inequality also holds. The universality of these theories predicts that the fluctuation-induced acceleration phenomenon found in kinesin is also applicable to general enzymatic reactions. Therefore, nonthermal fluctuations produced inside the cell may have the effect of activating general enzymes working inside the cell as a sort of *vitality of life*.

## Fluctuation-induced acceleration in other intracellular molecules

A similar acceleration phenomenon was reported for dynein, which transports vesicles on microtubules in the opposite direction to kinesin (Ezber et al. 2020). Unlike kinesin experiments, in which a constant load in the backward direction was applied while a random fluctuation was added, dynein experiments alternated external forces in the forward and backward directions. In this case, dynein accelerated beyond its maximum velocity at no average load. This behavior contrasts that of kinesin, whose maximum velocity changed little under small average loads. On the other hand, kinesin can quickly dissociate from a microtubule when a forward pulling force is applied (Milic et al. 2014). Furthermore, unloaded kinesin adopts a compact conformation with its heads attached to the tail to inhibit futile ATP hydrolysis (Coy et al. 1999; Friedman and Vale 1999; Kaan et al. 2011; Aoki et al. 2013). Therefore, it is thought that kinesin reduces its energy consumption by being passively transported away from the microtubule in response to external forces applied in the forward direction.

Fluctuation-induced acceleration is not limited to molecular motors. Enzymes generally undergo conformational changes during their reactions and follow the Arrhenius equation. Adding the universality of Jensen's inequality, it is expected that the reaction rate of any intracellular enzyme can be enhanced if fluctuations are incorporated in a successful way. Furthermore, it has been reported that not only enzymatic reactions, but also DNA loop formation (Chen et al. 2010) and protein folding rates (Tapia-Rojo et al. 2020) can increase due to fluctuations in the external environment. On a larger scale, several examples have already been reported where nonthermal fluctuations per se are utilized for physiological functions. For example, the random movements of mitochondria facilitate their even distribution during cell division (Moore et al. 2021), and the active fluctuations of membrane potential in paramecium contribute to the expression of spontaneity (Oosawa 2007). Just recently, the first verification for intracellular acceleration correlated to nonthermal fluctuations generated by actomyosin activity were reported in dynein (Torisawa et al. 2024). The next challenge is to identify enzymes (not just motor molecules) that are activated by utilizing nonthermal fluctuations in living cells.

Nonthermal fluctuations in a cell are generated by the metabolic activity of numerous kinetic molecules, which themselves consume a significant amount of energy. Consequently, the thermodynamic efficiency of single-molecule enzymes using environmental nonthermal fluctuations is low. However, the output work of molecular machines that utilize nonthermal fluctuations in the cell may itself be a source of these fluctuations (Hurst et al. 2021). To discuss the "efficiency" of molecules working in cells, quantitative criteria different from thermodynamic efficiency, such as optimization for transport efficiency (Hwang and Hyeon 2018) or fitness for the actual working environment (Kobayashi and Sughiyama 2015) at the molecular scale, are necessary.

## Summary

The interior of living cells is filled with nonthermal fluctuations that are spontaneously produced by energy consumption due to metabolic activity. These nonthermal fluctuations are suggested to affect the activity of kinesin and other general enzymes in cells (Ariga et al. 2021). However, the acceleration phenomenon *in general enzymes* due to intracellular nonthermal fluctuations is still only a prediction generalized by mathematical analysis based on limited in vitro experiments under a microscope (Ball 2021). Although the first example for the intracellular acceleration was just reported in dynein movement (Torisawa et al. 2024), further direct verification is needed to determine whether this phenomenon is generally utilized in living cells. Nevertheless, the combination of a bottom-up approach based on state-of-the-art microscopy, which reproduces mechanical interactions as an environment, and an analytical approach based on mathematical model simulations using universal theoretical equations will be highly beneficial for elucidating discrepant phenomena frequently encountered by cell biologists that are observed in cells but not reproduced in vitro (Ross 2016).

## Data Availability

No datasets were generated or analysed during the current study.
